# Multimodal Artificial Intelligence in Medicine

**DOI:** 10.34067/KID.0000000000000556

**Published:** 2024-08-21

**Authors:** Conor S. Judge, Finn Krewer, Martin J. O'Donnell, Lisa Kiely, Donal Sexton, Graham W. Taylor, Joshua August Skorburg, Bryan Tripp

**Affiliations:** 1 HRB-Clinical Research Facility, University of Galway, Galway, Ireland; 2 Insight Data Analytics, University of Galway, Galway, Ireland; 3 Department of Medicine, Trinity College Dublin, Dublin, Ireland; 4 University of Guelph, Guelph, Ontario, Canada; 5 Vector Institute, Toronto, Ontario, Canada; 6 Department of Systems Design Engineering, University of Waterloo, Waterloo, Ontario, Canada

**Keywords:** AKI

## Abstract

Traditional medical artificial intelligence models that are approved for clinical use restrict themselves to single-modal data (*e.g*., images only), limiting their applicability in the complex, multimodal environment of medical diagnosis and treatment. Multimodal transformer models in health care can effectively process and interpret diverse data forms, such as text, images, and structured data. They have demonstrated impressive performance on standard benchmarks, like United States Medical Licensing Examination question banks, and continue to improve with scale. However, the adoption of these advanced artificial intelligence models is not without challenges. While multimodal deep learning models like transformers offer promising advancements in health care, their integration requires careful consideration of the accompanying ethical and environmental challenges.

## Introduction

The collection and interpretation of multimodal data, including images (*e.g*., x-rays), unstructured text (*e.g*., clinical notes), and structured data (*e.g*., laboratory results), has been a critical component of caring for patients.^1,2^ Health care workers collect and combine these data, making informed diagnoses and treatment decisions, often using a semiquantitative approach. Although seasoned clinicians understand the inherently multimodal nature of diagnostic and therapeutic medicine, most current medical artificial intelligence (AI) models that are approved for clinical use are not multimodal,^3^ employing AI models that are specialized for one specific task and usually use one type of clinical data.^4^ Our article focuses on recent developments in multimodal deep learning applications in health care and discusses some of the risks and potential opportunities of this very fast-paced technologic revolution.

## Scenario 1—Multimodal Human Intelligence (Current Clinical Practice)

Mary is a 70-year-old woman who attends the emergency room with a persistent cough with green-colored sputum and fever. She has a background of mild heart failure (ejection fraction 45%), which has been stable for the past year. She has continued her routine medications, which are ramipril, frusemide, aspirin, and occasional nonsteroid anti-inflammatories. In the emergency department, routine blood tests revealed raised C-reactive protein (96) with normal renal and liver profile. Her CURB-65 score was 3, and she was admitted to hospital for community-acquired pneumonia. All preadmission medications are continued because there is no specific concern by the admitting team. By day 2 of admission, serum urea and creatinine were elevated, and she was diagnosed with an AKI. The team discontinued all medications with adverse renal effects, including ramipril, frusemide, and nonsteroidal anti-inflammatories. On day 5 of admission, Mary has an exacerbation of heart failure, which extends her hospital admission by a further 8 days because it is also associated with an episode of delirium.

## Scenario 2—Assistance of Single Modal AI

**Figure 1:**
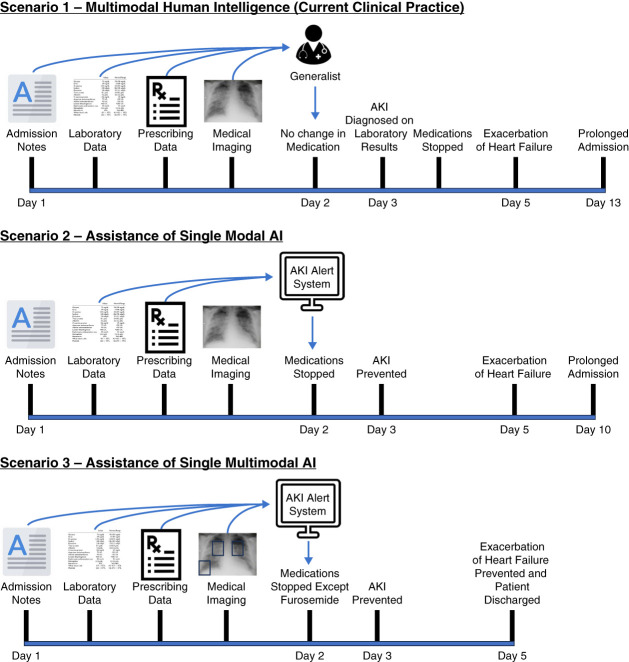
**Patient scenarios without AI, with single modal AI and with multimodal AI.** AI, artificial intelligence.

The acute hospital has recently introduced an AKI alert system that takes data from the electronic health record (laboratory results, diagnosis codes, vital signs, and medication records) and generates a predicted probability of developing AKI within 48 hours of admission (Figure 1).^5^ The AKI alert system employs a deep learning architecture called a recurrent neural network. The AKI alert system indicates a high probability of Mary developing an AKI and recommends holding all nephrotoxic medications (ramipril, frusemide, and nonsteroidal anti-inflammatories), despite normal renal laboratory results. This model does not have any information about her volume status (hypovolemia, euvolemia, or hypervolemia). Although Mary does not develop an AKI, she does have an exacerbation of acute heart failure with prolonged admission associated with delirium.

## Scenario 3—Assistance of Multimodal AI

**Figure 2:**
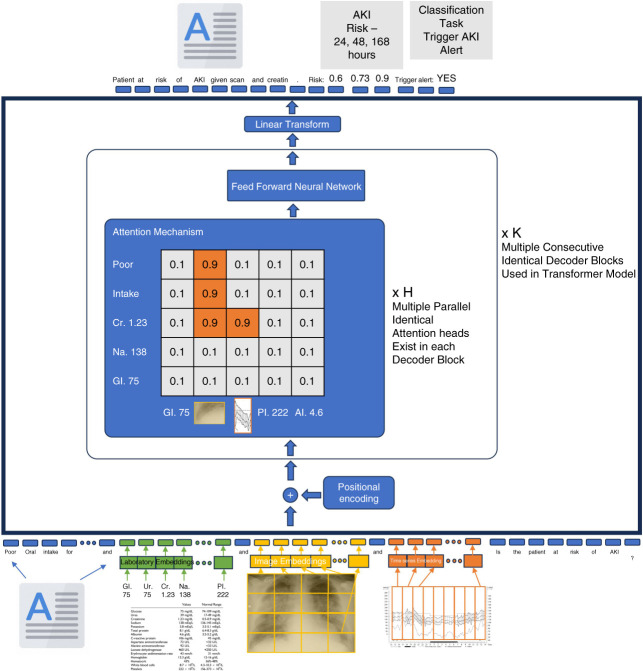
**A future multimodal transformer-based AKI alert system.** This figure depicts a multimodal transformer-based system designed to predict AKI risk by integrating text, structured data (*e.g*., laboratory tests), images (*e.g*., chest x-ray), and time series data. These are converted into numerical vectors through specific embedding layers. Positional encoding adds order information to these vectors, which are processed by the attention mechanism. The heatmap shows how the model focuses on relevant data parts, using keys, queries, and values to compute attention scores. Transformed vectors pass through a feed forward neural network for pattern learning. The architecture includes multiple identical decoder blocks for iterative refinement. At the top, the classification task predicts AKI risk at 24, 48, and 168 hours, triggering alerts if risk thresholds are exceeded. Cr, creatinine; Gl, glucose, Na, sodium; x H, attention heads; x K, decoder blocks.

The same acute hospital has recently introduced a multimodal AKI alert system that takes in multiple types of data (clinical notes, laboratory data, imaging request details, medical images, imaging reports, clinical measurements, and prescription records) (Figure 2). The multimodal AKI alert system is built using a deep learning architecture called a transformer model (Table 1). In addition to the predictive probability of AKI, the AI system also considers findings on chest x-ray, which reveals subtle features of mild pulmonary edema, along with a pneumonic infiltrate. In this scenario, the AI algorithm recommends holding ramipril and nonsteroidal anti-inflammatories, but to continue diuretic therapy. In this management scenario, the patient recovers from community-acquired pneumonia and discharged on day 3 of admission and does not experience AKI and exacerbation of heart failure or delirium.

**Table 1:** AI concepts and definitions

Concept	Description
Architectures	AI systems can be broken down into architectures, model families, models, and model instances. Common AI architectures include CNNs in image or pattern recognition tasks and transformers in sequence-to-sequence prediction and classification tasks. Various transformer model families have been developed, such as GPT and Flan-T5. Within model families, models are differentiated by their size or model improvements, such as Flan-T5-Small, Flan-T5-Base, and Flan-T5-Large or GPT-2 and GPT-4. Model instances refer to a specific set of weights trained in a model, often for a specific problem domain, such as Llama-2-13B-Instruct
Vectors	Vectors are essential components of transformers. A vector is a list of numbers. In natural language processing, words are converted into vectors to be processed by a machine learning model. These vectors are optimized to represent the semantic and syntactic characteristics of the words, allowing the model to understand and manipulate language. High-dimensional (long) vectors can capture more nuanced relationships and meanings than lower-dimensional ones.
Transformers	In a text-processing application, such as a chatbot, the input to a transformer is a sequence of words. The transformer converts each word into a vector, *i.e*., an ordered list of numbers. Once a word has been converted into a vector, the transformer repeatedly updates each vector with information from other vectors, corresponding to words that appear earlier in the sequence. It does this selectively using an attention mechanism (described below). This adds context to the current word. For example, at the end of the sequence, “…confirms the right ventricle is enlarged,” the attention mechanism might add together the vectors for “right,” “ventricle,” and “enlarged” to produce a new vector that means roughly “enlarged right ventricle.” Large transformers repeatedly combine and transform the inputs, ultimately producing a prediction about which word should come next. Mathematically, these combinations and transformations rely on matrix multiplications
Attention	The attention mechanism is based on the idea of key-value pairs, which are widely used in information systems. A hospital information system might use key-value pairs to keep track of which patient is in which bed, *e.g*., the bed Red-North-12 (a key) may have the patient with ID 12345 (a value). If a user provides a bed identifier (a query) that matches one of the keys, the system will provide the corresponding value (the patient ID). Transformer attention uses partial matches between queries and keys. This is roughly analogous to querying the bed system with Red-North and getting back a list of all the patients in that unit. In a transformer, the queries, keys, and values are vectors. The transformer creates a query vector by multiplying the current word's vector by a matrix (the matrix elements are learned network parameters). Similarly, it creates the keys and values by multiplying the previous words' vectors by different matrices. It calculates partial matches according to the similarity between the query vector and all the key vectors. The transformer then combines the value vectors that correspond to any partially matching keys—not as a list, but as a weighted sum. This is how previous sequence elements are combined to add context and other nuance to the current word vector
Encoding	Encoding in the context of transformers refers to the process of converting input data (like text, images, sound) into a numerical format that can be processed by the model. In NLP, this often involves converting words (or part of words) into vectors. Encoding is a critical step in preparing data for complex tasks, such as medical question answering, report generation, and medical summarisation

AI, artificial intelligence; CNN, convolution neural network; GPT, generative pretrained transformer; NLP, natural language processing.

## Transformer AI Models (Origins)

To appreciate the anticipated role of transformer models in clinical care, it is useful to consider their origins. A transformer is an AI deep learning model architecture (Table 1) which is predominately used to process information provided in a sequence, and they were first used for translating sentences from one language to another.^6^ In addition to translating individual words, a transformer model captures the context of each word with interpretive relevance to all other words in the sentence, a process known as attention (Table 1). The transformer architecture is the foundation of complex large language models (LLMs), which use extensive and interconnected transformer models in the interpretation of information from diverse sources. Following interpretation, transformer models can provide summary interpretation to the human user. Such models are referred to as generative LLMs (*e.g*., generative pretrained transformer [GPT] family of models). Generative LLMs are trained on extensive amounts of general text data (Internet web pages, books, and Wikipedia), allowing them to generate human-like text and complete complex language tasks,^7–9^ resulting in the ability to provide detailed answers to specific questions.

### Transformers/LLMs Go to Medical School

As the sophistication of transformer models to acquire and interpret information grew, an obvious target for deployment was health care delivery. We might consider the evolution of LLMs to the development and training of a medical doctor. An initial step, therefore, is the acquisition and assimilation of medical information, suitable to correctly answer knowledge-based medical questions. Several LLMs have undertaken training (studying) in large datasets of medical information and then tested their knowledge in conventional medical examinations. For example, BioMedLM, trained on biomedical literature from PubMed, achieved a score of 50.3% in medical question answering on the MedQA benchmark, which is a USMLE-style question bank (December 2022).^10^ Medical pathways language model (Med-PaLM) 2 is a closed source medical transformer model that was trained on general data and then fine-tuned on medical data.^11^ Med-PaLM 2 has reached a score of 86.5% on the MedQA benchmark (June 2023)^11^ and GPT-4, also closed source, has surpassed 90%.^12^ Open-source models are also quickly reaching this standard: Meditron-70B is a smaller open-source model which achieved a score of 70.2% on MedQA (November 2023).^13^ Currently, LLMs can successfully complete knowledge-based assessments of medical knowledge.

### Transformers/LLMs Can Be Bad Students

LLMs often produce incorrect hallucinated text, such as fabricating information in responses, a behavior not uncommon among medical students.^14^ The Galactica LLM by Meta^15^ was trained on high-quality sources, including medical textbooks and journal articles, and provided an interactive interface. However, users discovered that it produced false output with an authoritative tone and convincing detail, such as well-written journal articles on the benefits of eating crushed glass.^16^ Such tendencies arise from the way LLMs learn to model language.^17^ Training generative transformer models mostly consists of learning to predict the next word in human-written texts (autoregressive training). For example, the words “presented with urinary frequency and 48 hours of fever and rigors. He was hypotensive and” are likely to be followed by “tachycardic.” To some extent, LLMs encode this underlying reality (Figure 2).^18^

LLMs may output fabrications, such as inserting a plausible body mass index into a generated medical note despite lacking access to the patient's weight.^19^ As such, the LLM may provide a best guess rather than accurate information but not reflect uncertainty in the response. To improve the accuracy of responses, a process of supervised fine-tuning involves a labeled dataset of prompts and desired responses written by humans to fine-tune the responses that the model gives.^20^ Alignment training of GPT models uses reinforcement learning with reward-based human feedback on the basis of human-interpreted quality of output.^21^ Such methods have greatly improved GPT models, but confabulation persists. A recent study reporting the use of ChatGPT in an intensive care unit setting found that the answers provided by ChatGPT were often erroneous or dated, and the output would often contain incorrect factual pieces of information like dates and doses of medications.^22^ A further challenge is that LLMs are unable, currently, to distinguish which outputs are based on factual information and which are based on hallucinations.

## Transformers Work Effectively with Any Modality of Data—Medical Data Interpretation

Transformers, like other neural network architectures, require numeric input. To enable a transformer to read text, the words must be converted into a list of numbers called a vector through a process called encoding (Table 1). Beyond that first step, nothing about the operation of the transformer model is specific to language. The same network structures and training methods work well with entirely different modalities of data, such as sounds and images. Vision transformer models, such as Medical Segment Anything Model, achieved state-of-the-art performance on many medical image segmentation tasks over preexisting specialized nontransformer models.^23^ Transformers also excel in audio processing tasks, such as classifying sounds^24^ and transcribing speech.^25–28^ Recently, transformers have been applied to bio signals as well. For example, strong performance has been reported in the classification of arrhythmias and epileptiform activity, with transformers that were trained on large unlabeled electrocardiogram and electroencephalogram datasets and fine-tuned on smaller classification datasets.^29^ Where direct comparisons have been made, transformers often outperform other kinds of networks, such as convolutional neural networks, if they are first trained on large volumes of data^30^ or on tasks analogous to next-word prediction.^25,29,30^ Transformers need large volumes of unlabeled data (*e.g*., images only), after which they can quickly learn a particular task with limited labeled data (*e.g*., image-category pairs).

## Multimodal Transformers—Interactive Medical Cases

Transformers are often trained to process multiple modalities. The DALL-E image generation model was trained on hybrid sequences consisting of image descriptions, followed by image patches.^31^ Given only an image description, it could predict, patch-by-patch, an image likely to match the description.

Multimodal transformers like GPT-4 and Gemini can describe and reason about images. The company Be My Eyes is using this technology to develop a smartphone app for visually impaired people which can describe and answer questions about their visual surroundings.^32^ At the time of publication (July 2024), the image-related capabilities of GPT-4 Vision were evaluated on *The New England Journal of Medicine* (88.7%) and *JAMA* (73.3%) image challenges, consistently outperforming human readers.^33^ A technical study released by OpenAI tested the ability of GPT-4V to give medical advice^34^ reported serious interpretation errors for medical imaging, including misdiagnosing the lateralization in medical images.

Med-PaLM Multimodal is a large milestone toward a generalist biomedical AI system. It encodes and interprets multimodal biomedical data, including clinical language, imaging, and genomics with the same set of model weights.^35^ It required a new testing benchmark (MultiMedBench) to be curated and includes multimodal medical tasks, such as radiology and pathology visual question answering and dermatology and mammography image classification, for which Med-PaLM Multimodal reached state-of-the-art performance.

In radiology, embeddings for language/image-aligned x-rays is an approach that combines LLMs with vision encoders and was trained on the medical information mart for intensive care – chest x-ray dataset that contains pairs of chest x-ray images and chest x-ray text reports.^36^ Embeddings for language/image-aligned x-rays demonstrated state-of-the-art classification performance comparable with other supervised learning methods on complex visual questions.

Health LLM (HeLM) combines high dimensional clinical information with text prompts to estimate disease risk.^37^ This approach, on the basis of the pathways language model - multimodal edition model, trains an encoder which maps high dimensional data into the LLM's token embedding space. Using text and encoded tabular data from the UK Biobank HeLM predicts all-cause mortality and hypertension with superior accuracy to traditional machine learning algorithms using the same dataset. The HeLM model demonstrates improved accuracy in predicting asthma phenotype when combining spirometer data with text prompts and encoded tabular data over text prompt and tabular data only.

## Transformers Improve Reliably with Model and Data Scale—Clinical Experience

While there have been important technical advances in recent years, *e.g*., to allow LLMs to carry on longer conversations,^38–40^ today's transformers are structured in much the same way as older transformers, except that they are larger in every dimension. By some measures, the performance of LLMs improves predictably with scale.^9,41–43^ Other abilities, like mathematical operations, seem to emerge suddenly at certain model scales.^44,45^ The sudden emergence of a capability might say more about the task than the model, in that the success measure may be a nonlinear function of a deeper capacity that emerges more gradually.^46^

Transformers are much less sophisticated than the human brain, so one might suspect that they will not surpass humans without significant changes. However, they have advantages of scale. Some transformers have detailed working memory for hundreds of pages of text, whereas human working memory begins to struggle with any more than a phone number. PaLM^47^ was trained on 780 billion tokens (roughly 585B words), corresponding to roughly 10,000 years of full-time reading for a human. Later stages of LLM training currently rely on humans to rank their responses. However, LLMs seem to be even better at critiquing than producing answers,^19^ suggesting a potential path to indefinite improvement. The most imminent limiting factor is that there is only so much high-quality text available to consume. Recent transformers have already been trained with perhaps one-tenth of the total high-quality text that exists.^48^

## Future Directions and Risks for Multimodal AI in Health Care

Another set of considerations are whether current ethical and legal frameworks are adequate to address the challenges raised by multimodal AI in health care. The past decade has seen a burgeoning literature develop on the ethics of AI in health care generally. Issues, such as data ownership, informed consent, bias, privacy, responsibility gaps, opacity, digital divides, and environmental impact, have been extensively studied.^49^ Rapid LLM development and lag time in academic publishing result in few studies on LLM ethics in health care.^50–54^ High-level ethical principles often lack actionable guidance in specific medical AI contexts. Thus, we think that recent approaches which focus on stakeholder engagement and context-specific analysis of potential harms^55–57^ strike the right balance between realizing the transformative potential of AI, while avoiding ethical pitfalls.

Importantly, large models could be detrimental to health, even if they benefit health care. LLMs use large amounts of electricity and could drive significant CO_2_ emissions, exacerbating potential health impacts of climate change.^58,59^ Large model training is carbon-intensive, *e.g*., training GPT-3 produced about 552 metric tons of CO_2_-equivalent emissions. However, most emissions tend to come from use of the model after training.^60^ Future CO_2_ emissions depend on model sizes, hardware, the source of electricity used, and algorithm improvements,^60–63^ but there is a risk of substantial impact. For instance, processing each health data item once, a model like PaLM^47^ requires as many floating point operations as it has parameters (540B). Health data may currently be growing by about 1.5×10^21^ bytes per year.^64^ Assuming for simplicity that much health data consists of 16-bit images,^30^ this would correspond to roughly 3×10^18^ tokens per year. If this processing were done on typical current hardware, such as Nvidia A100 graphics processing units, using power from the eastern United States, which is moderately carbon-intensive,^61^ this would result in about 3.5×10^10^ metric tons of CO_2_ emissions per year,^47^ roughly 10% of the current total for health care.^65,66^

Environmental impacts are just one of several categories of AI risk.^63^ Large models may also pose various extreme risks that are difficult to mitigate.^67^ For example, it is hard to rule out the possibility that a future model could have ill intent toward humans (perhaps due to a malicious developer or as an unexpected side effect) that it hides until it is widely deployed.^67^ In addition, it is important to avoid a scenario in which health care's legitimate needs and large market motivate AI developers to develop capabilities that ultimately prove detrimental to health overall. The weighing of risks and benefits to health should be performed by health care providers, policy makers, and independent researchers, rather than by companies that stand to profit more from one outcome than another (Table 2).

**Table 2:** Opportunities and challenges of multimodal artificial intelligence applied to health care

Opportunities	Challenges	Solutions to Mitigate Challenges
Effectively process and interpret various data types, such as text, images, and structured data	Data ownership and privacy	Implement robust data governance frameworks and ensure informed patient consent
Enhanced diagnostic and predictive capabilities because of integration of additional data from diverse data types	Transparency	Create trust in medical AI systems through the application of rigorous randomized controlled trials^68^ and involvement of relevant stakeholders^56,57^
Early detection of diseases	Increasing inequity	Low- and middle-income countries continue to be underrepresented in datasets and access to resources and compute. Continue working with communities to locate compute in those developing economies so that they can benefit from the AI advances
Excellent performance on standard benchmarks	Environmental impact	Prioritize energy-efficient model architectures. Choose cloud services that use renewable energy sources
Continuous improvement with scale	Hallucinations and errors	Incorporate guardrails when developing and deploying models.^69^ This is an active research area,^70,71^ so it will be important for system implementers to remain up to date on developments
Integration into clinical workflows	Integration into clinical workflows	Both an opportunity and a challenge. Requires alignment with existing practices, overcoming resistance to change among health care professionals, and especially training health care professionals how to use AI systems safely and effectively^72^

AI, artificial intelligence.

## Conclusion

In conclusion, the rapid evolution of LLMs and their multimodal counterparts presents both promising opportunities and formidable challenges for health care. The expanding capabilities of these AI models, exemplified by GPT-4 and Med PaLM 2, demonstrate remarkable advancements in tasks ranging from medical question answering to medical image reporting. The integration of these models in areas like radiology and disease prediction has shown potential for significant improvements in clinical outcomes. However, this technologic progress is not without its ethical, legal, and environmental ramifications. Furthermore, the considerable environmental impact of these models, particularly in terms of carbon emissions, raises concerns about their sustainability and the indirect health consequences of climate change.

The future of health care will undoubtedly be shaped by multimodal AI, and it is our collective responsibility to steer this course with a commitment to the betterment of human health.
